# Supplementary material for: Higher Epoxyeicosatrienoic Acids in Cardiomyocytes-Specific CYP2J2 Transgenic Mice Are Associated with Improved Myocardial Remodeling
Source: Biomedicines. 2020 May 30;8(6):144. doi: 10.3390/biomedicines8060144 (PMC7344501; doi:10.3390/biomedicines8060144)
Supplement: Supplementary file 1 [file biomedicines-08-00144-s001.pdf]

SUPPLEMENTARY MATERIALS

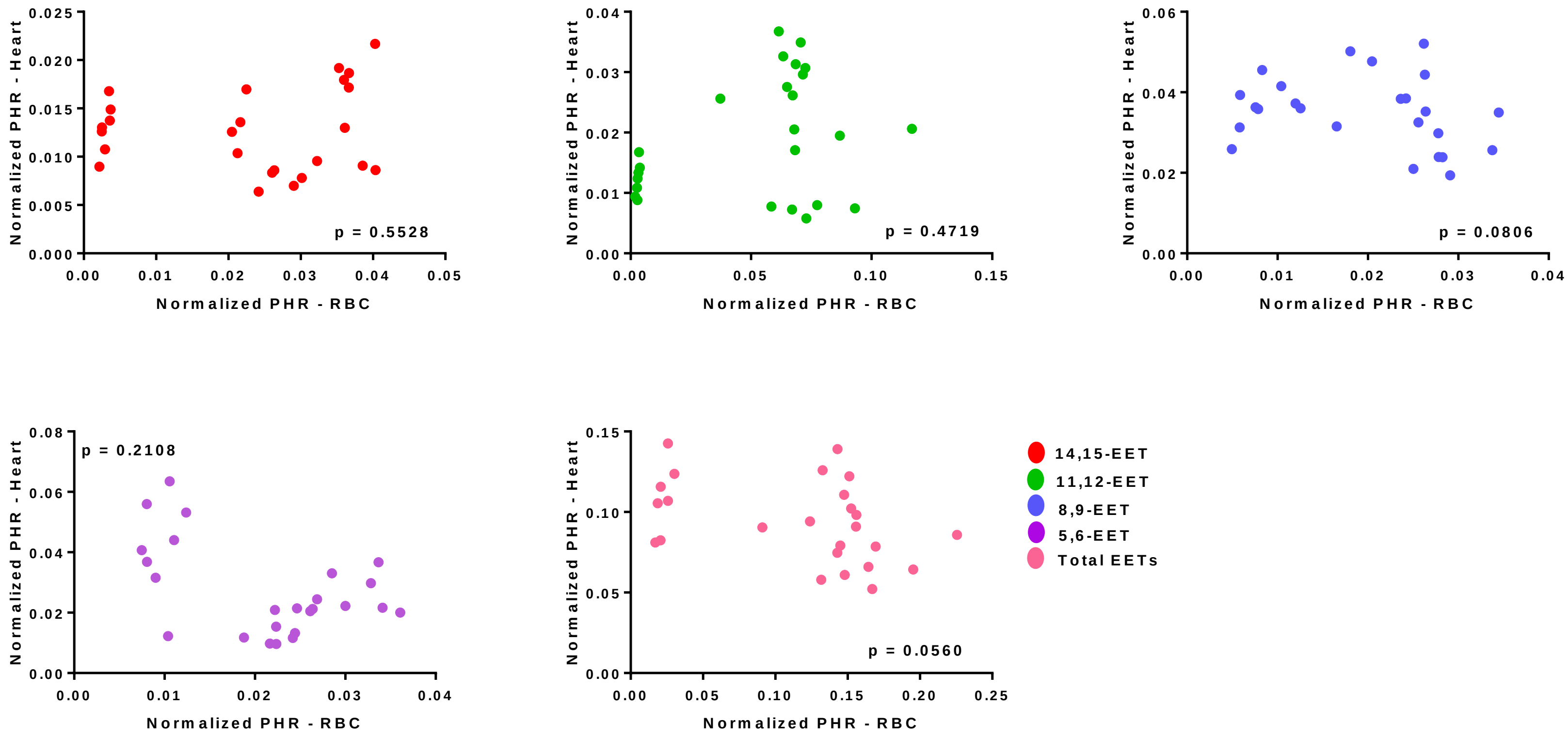

Supplementary Figure 1. Correlation of each regioisomer of *trans*-EET and total *trans*-EETs in erythrocyte membrane and cardiac tissue of WT mice.

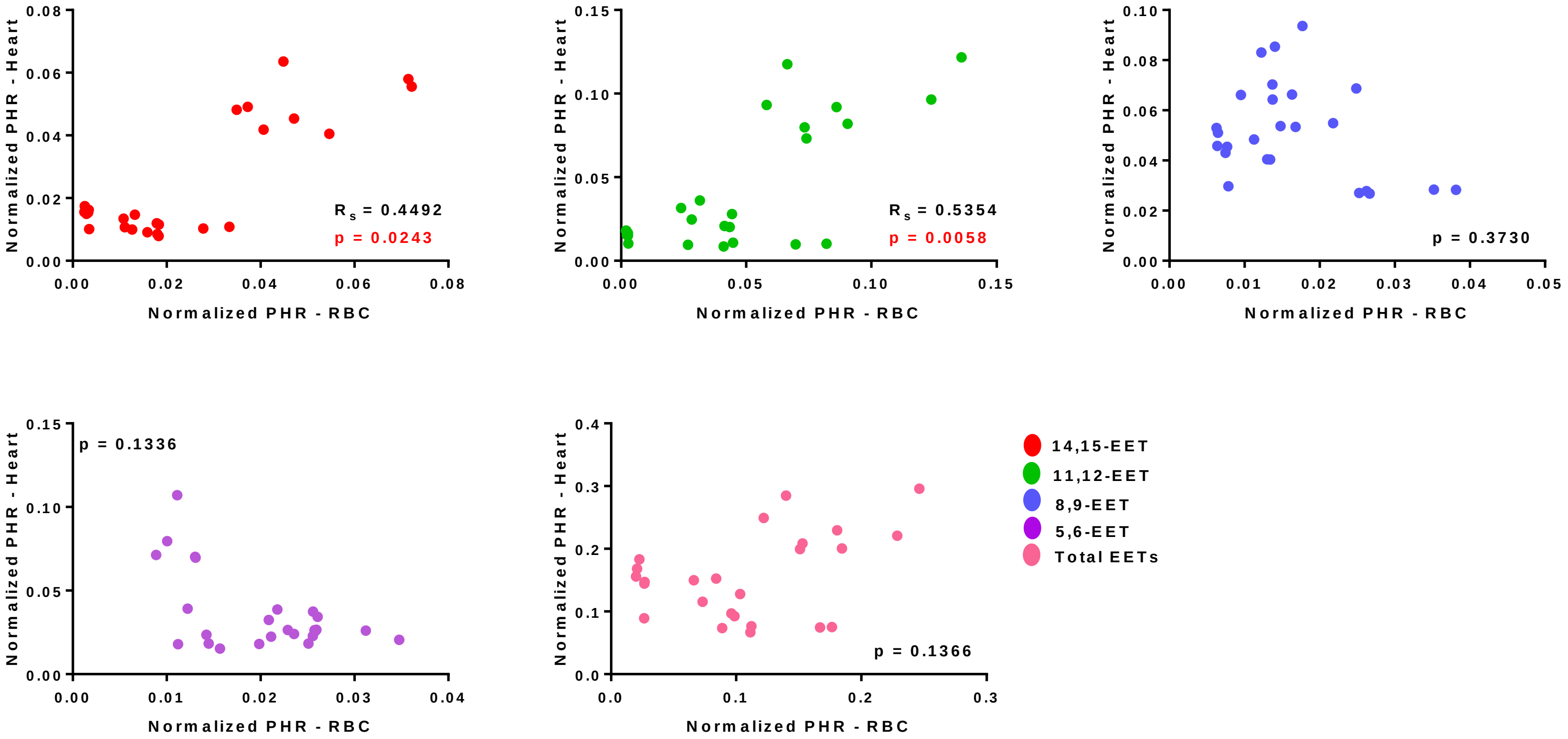

Supplementary Figure 2. Correlation of each regioisomer of *trans*-EET and total *trans*-EETs in erythrocyte membrane and cardiac tissue of Tr mice.

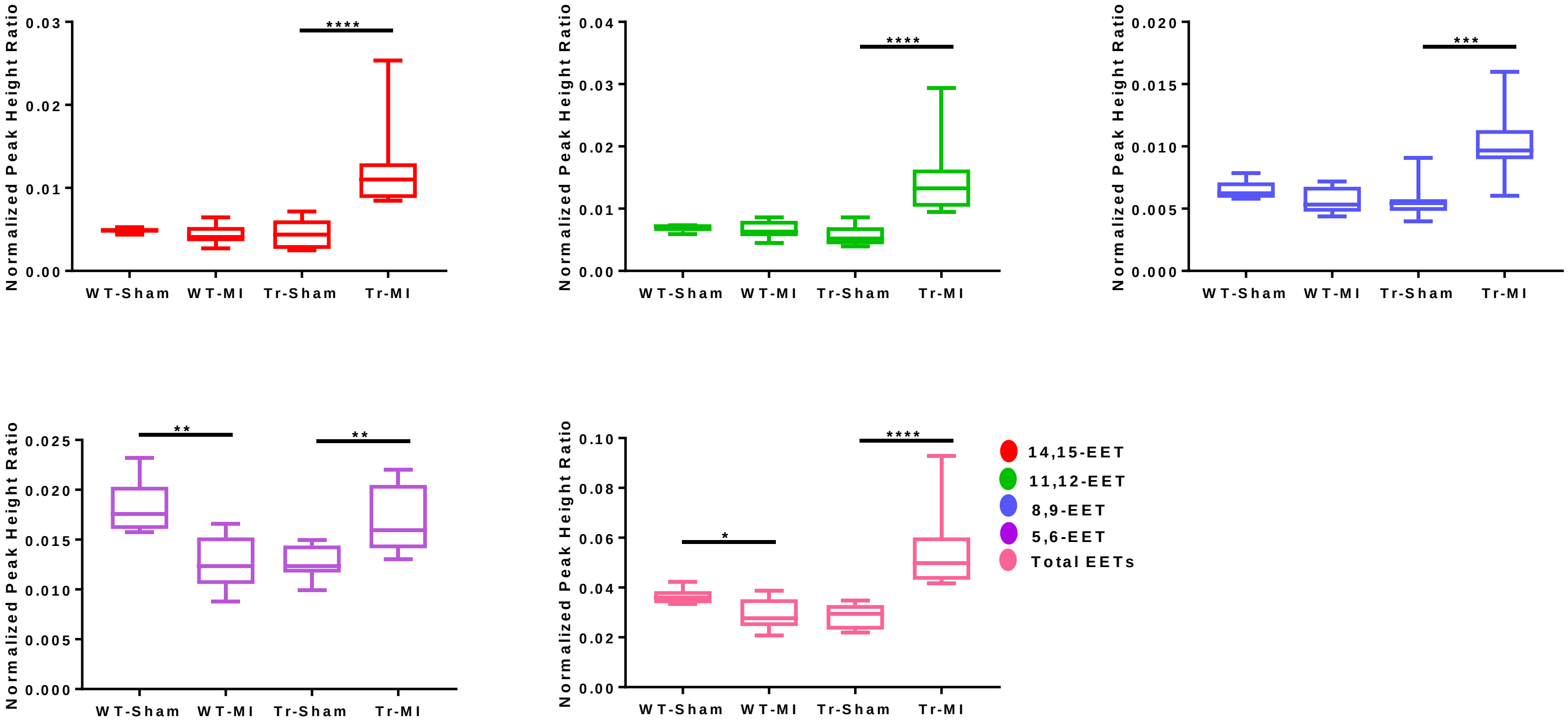

Supplementary Figure 3. Levels of each regioisomer of *trans*-EETs and total *trans*-EET extracted from erythrocyte membrane of WT and Tr mice subjected to sham or MI surgery. * indicates $p \leq 0.05$, ** indicates $p \leq 0.01$, and **** indicates $p \leq 0.0001$.

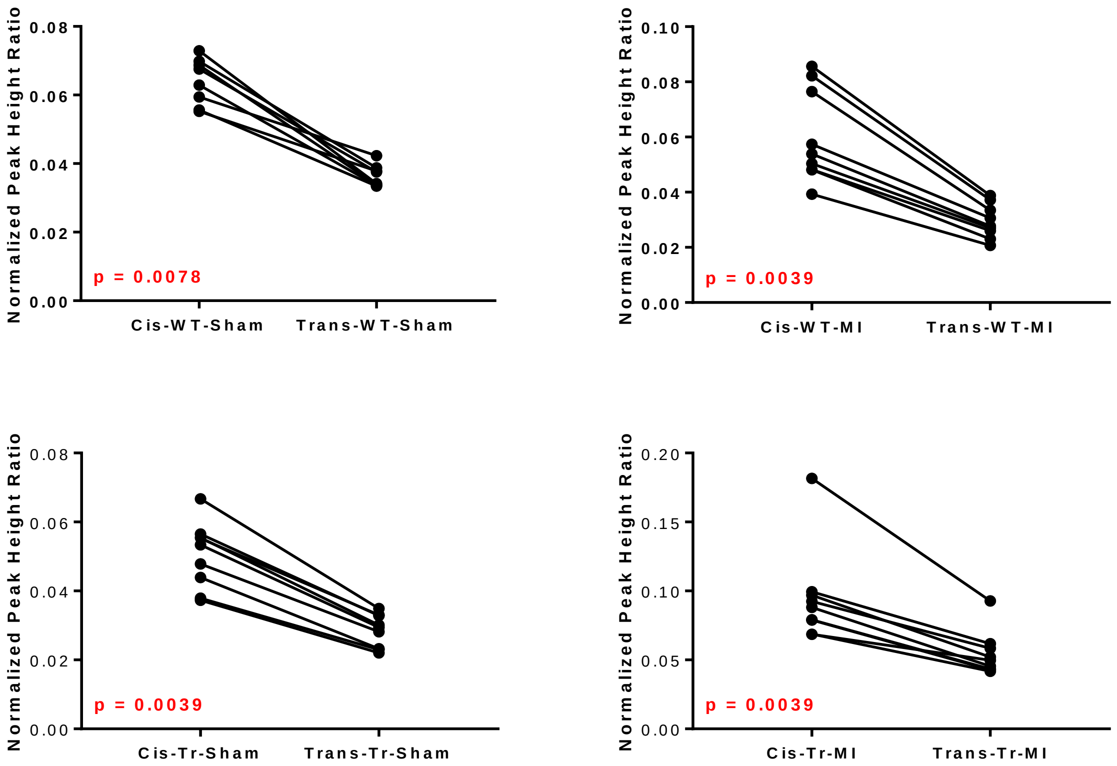

Supplementary Figure 4. Comparison between total *cis*- and *trans*-EETs levels extracted from erythrocyte membrane of WT and Tr mice.

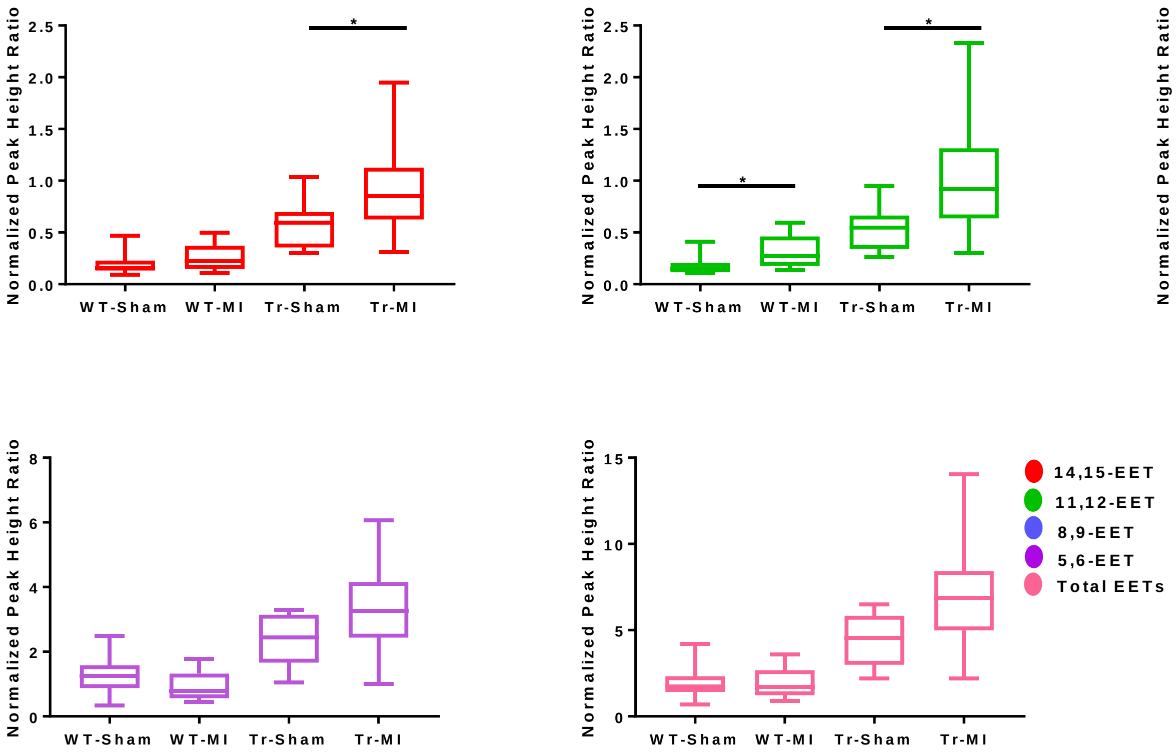

Supplementary Figure 5. Levels of each regioisomer of *trans*-EETs and total *trans*-EETs extracted from cardiac tissue of WT and Tr mice subjected to sham or MI surgery. * indicates $p \leq 0.05$.

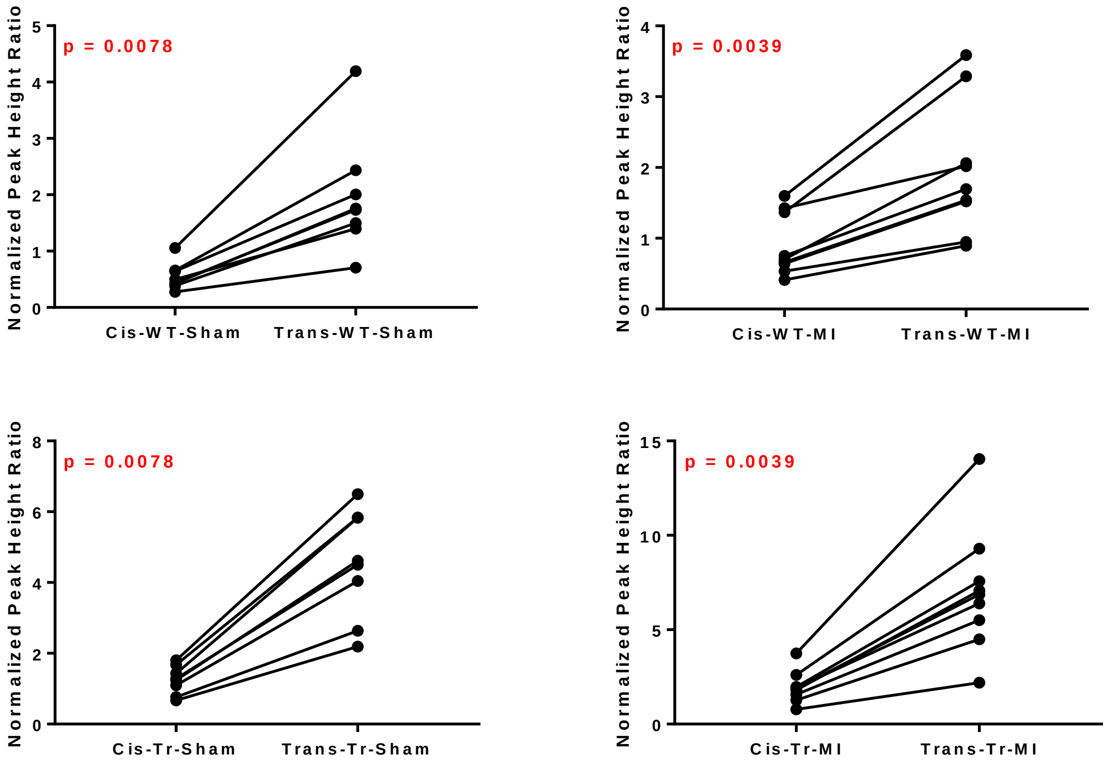

Supplementary Figure 6. Comparison between total *cis*- and *trans*-EETs levels extracted from cardiac tissue of WT and Tr mice.

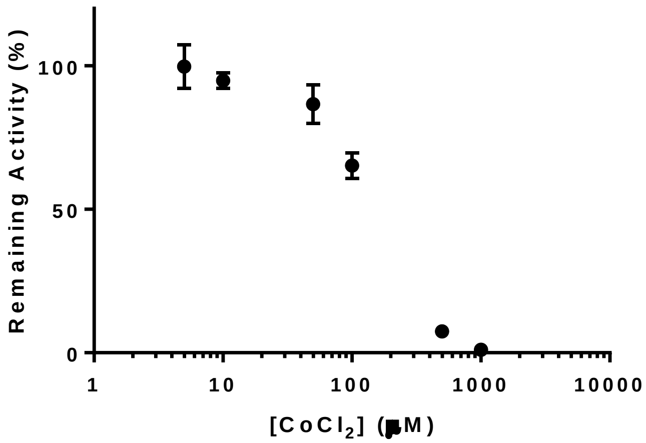

Supplementary Figure 7. CYP2J2 activity in human adult ventricular myocytes measured using terfenadine as a probe substrate in the presence of varying concentration of CoCl2. Each point is the average of triplicates, and error bars represent the standard deviation of replicates. Activity begins to drop in the presence of 50 μM CoCl2 (15% drop in activity), with greater loss in activity at higher concentrations.
